# NS5806 Induces Electromechanically Discordant Alternans and Arrhythmogenic Voltage-Calcium Dynamics in the Isolated Intact Rabbit Heart

**DOI:** 10.3389/fphys.2019.01509

**Published:** 2019-12-20

**Authors:** Sufen Wang, Moisés Rodríguez-Mañero, Sergio H. Ibarra-Cortez, Bahij Kreidieh, Laura Valderrábano, Majd Hemam, Liliana Tavares, Elvin Blanco, Miguel Valderrábano

**Affiliations:** ^1^Department of Cardiology, Houston Methodist DeBakey Heart and Vascular Center, Houston Methodist Hospital, Houston Methodist Research Institute, Houston, TX, United States; ^2^Cardiology Department, Complejo Hospital Universitario de Santiago, Santiago de Compostela, Spain; ^3^Instituto de Investigación Sanitaria (IDIS), Universidad de Santiago de Compostela, Santiago de Compostela, Spain; ^4^Centro de Investigación Biomédica en Red de Enfermedades Cardiovasculares (CIBERCV CB16/11/00226 - CB16/11/00420), Madrid, Spain; ^5^Department of Nanomedicine, Houston Methodist Research Institute, Houston, TX, United States

**Keywords:** NS5806, Brugada syndrome, alternans, calcium cycling, ventricular fibrillation

## Abstract

**Background:** NS5806 activates the transient outward potassium current *I*_to_, and has been claimed to reproduce Brugada Syndrome (BrS) in ventricular wedge preparations. *I*_to_ modulates excitation-contraction coupling, which is critical in alternans dynamics. We explored NS5806-arrhythmogenic effects in the intact whole heart and its impact on alternans.

**Methods:** Langendorff-perfused rabbit hearts (*n* = 20) underwent optical AP and Ca mapping during pacing at decremental cycle lengths (CL). Spontaneous arrhythmias and pacing-induced alternans was characterized at baseline (BL), after perfusing with NS5806, before and after adding verapamil (VP), and SEA0400 (SEA, *n* = 5 each), to modulate Ca-current and Na-Ca exchange, the main AP-Ca coupling mechanisms.

**Results:** NS5806 induced BrS-like ECG features in 6 out of 20 hearts. NS5806 prolonged steady-state (3 Hz) action potential duration (APD) by 16.8%, Ca decay constant by 34%, and decreased conduction velocity (CV) by 52.6%. After NS5806 infusion, spontaneous ventricular ectopy (VE) and AP/Ca alternans occurred. Pacing-induced alternans during NS5806 infusion occurred at longer CL and were AP/Ca discordant from its onset. Spatially discordant alternans after NS5806 infusion had non-propagation-driven nodal line distribution. No spontaneous phase-2 reentry occurred. Under NS5806 + VP, alternans became AP/Ca concordant and only induced in two out of five; NS5806 + SEA did not affect alternans but suppressed spontaneous ectopy.

**Conclusions:** NS5806 disrupts AP-Ca coupling and leads to Ca-driven, AP/Ca-discordant alternans and VE. Despite BrS-like ECG features, no spontaneous sustained arrhythmias or phase-2 reentry occurred. NS5806 does not fully reproduce BrS in the intact rabbit heart.

## Introduction

NS5806, an activator of the transient outward potassium channel, *I*_to_, has been used as an experimental model of Brugada Syndrome (BrS) in both right and left ventricular wedge preparations (Calloe et al., 2009; Minoura et al., 2013) alone or in combination with verapamil (VP) (Szel et al., 2013; Szel and Antzelevitch, 2014; Patocskai et al., 2016, 2017; Yoon et al., 2018). Data were presented from a wedge preparation illustrating that heterogeneous loss of the action potential (AP) dome (plateau) at some, but not all, epicardial sites, creates an epicardial or transmural dispersion of repolarization, which induces phase-2 reentry and is responsible for arrhythmogenesis. A more recent study, however, reported that artifactual transmural AP gradients may have been induced by the wedge preparation (Boukens et al., 2017). A demonstration of NS5806 arrhythmogenic effects in the intact whole heart has been lacking.

The mechanisms of BrS arrhythmogenesis remain a subject of debate. Although primarily due to genetic mutations that reduce expression or function of cardiac Na channels [mutations in *SCN5A* (Chen et al., 1998), *CACNa1c*, *GPD1L*, *MOG1*, *SCN10A* (Bezzina et al., 2013), and plakophillin-2 (Cerrone et al., 2014)], *I*_to_ – most prominent in the right ventricle (RV) epicardium – is thought to cause its phenotypic expression. Epicardial shortening of the action potential duration (APD) due to unopposed *I*_to_ leads to transmural repolarization gradients that give rise to the characteristic ECG changes (Calloe et al., 2009) and set the stage for phase-2 reentry and ventricular fibrillation (VF) (Yan and Antzelevitch, 1999). The role of *I*_to_ in BrS arrhythmogenesis seems to be confirmed by the fact that gain-of-function mutations in *KCND3*-encoded Kv4.3 potassium channel, which generates *I*_to_- lead to BrS (Giudicessi et al., 2011), and, thus, the creation of NS5806, were shown to reproduce the ECG phenotype and arrhythmogenesis of BrS in canine wedge preparations (Calloe et al., 2009; Minoura et al., 2013). Increased *I*_to_ is also thought to play a role in arrhythmogenesis of early repolarization syndrome (Antzelevitch, 2013).

Modulation of *I*_to_ can alter APD alternans dynamics. T-wave alternans – the electrocardiographic manifestation of APD alternans – is a recognized harbinger of malignant ventricular arrhythmias and sudden cardiac death (Holley and Cooper, 2009). Alternans can be caused by APD restitution dynamics (voltage-driven) or by Ca-cycling dynamics (Sato et al., 2006), each with its own characteristics (Sato et al., 2006). Alternans occurs in BrS (Morita et al., 2006), and is a predictor of an increased risk of ventricular arrhythmias (Uchimura-Makita et al., 2014).

The *I*_to_ plays an important role in excitation-contraction coupling due to its effects on repolarization and on the L-type Ca current (*I*_Ca,L_) (Sah et al., 2003). Thus, it can have significant impact on Ca-cycling dynamics, which in turn can be arrhythmogenic *via* multiple mechanisms, including the generation of alternans (Liu et al., 2015). We tested the validity of NS5806 as a BrS model and its arrhythmogenic effects on an isolated intact whole rabbit heart model and focused on AP-Ca coupling.

Our data do not support NS5806 as an all-around BrS model, but demonstrate Ca-driven alternans during NS5806 infusion and suggest Ca-cycling arrhythmogenesis can occur *via I*_to_ modulation.

## Methods

All animal experiments have been approved by our Institutional Animal Care and Use Committee, and all experiments were performed in accordance with the Guide for Care and Use of Laboratory Animals (NIH Publication No.85-23, revised 2011).

**Figure 1:**
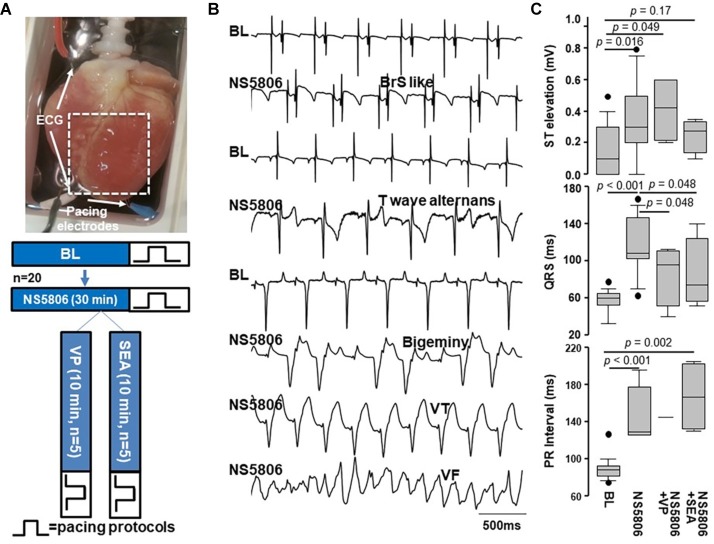
Experimental protocol and representative examples of ECG changes induced by NS5806. **(A)** Langendorff-perfused rabbit hearts with ECG and pacing electrodes (**top**) and experimental protocol (**bottom**). Hearts were perfused with Tyrode’s solution. After stabilization and BL recording, hearts were perfused with NS5806 first. Additional drug studies followed: VP and SEA. **(B)** Representative examples of ECG changes induced by NS5806: BrS-like ECG features, bigeminy, T-wave alternans, VT and VF episodes. All were non-sustained. VF episodes were only observed with pacing. **(C)** ST segment elevation, QRS duration, and PR interval in different conditions. NS5806 induced QRS widening, ST-segment elevation, and PR prolongation. NS5806 + SEA prolonged PR interval. NS5806 + VP increased ST elevation. Box = 25th and 75th percentiles. Bars = minimum and maximum values. Dots = outliers. *p* < 0.05 was considered statistically significant. BL, Baseline; VP, Verapamil; SEA, SEA0400.

Adult New Zealand white rabbits, 3–4 kg, were anesthetized with 50 mg/kg i.p. pentobarbital sodium and anticoagulated with 1,000 U/kg i.p. heparin. Hearts were rapidly removed and retrogradely perfused in a Langendorff apparatus (see Figure 1A) with a constant perfusion pressure of 60–65 mmHg at 37°C with oxygenated Tyrode’s solution containing (mM): NaCl 136, KCl 5.4, CaCl_2_ 1.8, MgCl_2_ 1.0, NaH_2_PO_4_ 0.33, Hepes 10, glucose 10, pH adjusted to 7.2 with NaOH. The perfusion pressure was monitored using an in-line physiological pressure transducer connected to the Bridge Amplifier (AD Instruments Inc., Colorado Springs, CO). ECGs were recorded using the Animal Bio Amplifier (AD Instruments Inc.) from perfusion start through to the experiment end using two wire leads, one connected to the right atrium and the other connected to the right ventricular apex. Data analyses were performed off-line using LabChart software (AD Instruments Inc.).

We used the Ca fluorophore Rhod-2 AM (0.2 mg dissolved in 0.2 ml of dimethyl sulfoxide containing Pluronic F-127, 20% wt/vol) to track intracellular Ca. This solution, diluted in 150 ml of Tyrode’s solution to achieve a final Rhod-2 concentration of 1.18 μmol/L, was infused into the heart over a 30-min period. The heart was then stained with voltage-sensitive dye RH237 (0.33 μM) for 10 min to measure membrane potential. Both dyes were from Invitrogen (Carlsbad, CA). Blebbistatin at a final concentration of 5 μmol/L was applied as an excitation-contraction uncoupler to eliminate motion artifacts. Studies have shown that blebbistatin at 5–10 μM completely eliminated contraction in all cardiac preparations but did not have any effect on electrical activity, including ECG parameters, atrial and ventricular effective refractory periods, and atrial and ventricular activation patterns. Blebbistatin at 10 μM had no effect on action potential morphology and intracellular calcium transients’ morphology (Fedorov et al., 2007; Lou et al., 2012). Fluorescence was excited with a LEX2 LED light source (532 nm, Brainvision Inc., Japan). Emitted fluorescence was split by a 630-nm dichroic mirror and collected using two electron-multiplying CCD cameras (Cascade 128+, Photometrics, Tucson, AZ). We used a grid to calibrate the locations of the field of view of the two CCD cameras. Using this calibration, we could compare the recordings of intracellular Ca and membrane potential from the same locations. For intracellular Ca, reflected fluorescence was collected through a 585 ± 20-nm filter. For membrane voltage, passed fluorescence was collected through a 710-nm long pass filter. Simultaneous signals were recorded with synchronized CCD cameras operating at 200 frames per second with a spatial resolution of 0.172 × 0.172 mm^2^ per pixel.

Hearts were paced at 2, 3, 4, and 5 Hz, up to 10 Hz from the apex using a square pulse stimulator (S88X, Grass Technologies, West Warwick, RI) before and after applying each drug. Pacing rates were variably increased up to a maximum rate that maintained 1:1 capture. Ten stimulations were applied in each pacing rate. Ca and action potential (AP) signals were recorded during and after pacing in totals of 5 s. Pacing at increasing rates was used to induce alternans. When induced, the alternans phases in APD and Ca transients were compared to assess electro-mechanical (AP/Ca) concordance (if short APD beats had small Ca transients) vs. discordance (short APD beats had large Ca transients). Additionally, the alternans phase (of APD and Ca) was assessed across the mapped heart surface for spatial concordance vs. discordance (Sato et al., 2006; de Diego et al., 2008). Pacing rates were progressively increased to assess dynamic behavior of alternans. APD and Ca transient alternans maps were constructed by subtracting APD or Ca transient amplitude, respectively, from consecutive beats in each pixel and color-coding the differences (de Diego et al., 2008).

### Drug Studies

NS5806 (Tocris Bioscience, Minneapolis, MN) was dissolved in dimethyl sulfoxide (30 mM stock) and stored at −20°C in aliquots, and freshly diluted in Tyrode’s solution at a final concentration of 5, 15, and 45 μM before use. Unless otherwise indicated, recordings at 45 μM NS5806 are shown. Verapamil (VP, Verapamil HCl Injection, USP 5 mg/2 ml, Hospira, Inc., Lake Forest, IL) at 2.5 μM final concentration was directly diluted from a stock solution of 2.5 mg/ml. SEA0400 (SEA, Tocris Bioscience, Minneapolis, MN) was dissolved in dimethyl sulphoxide (2 mM stock) and stored at −20°C in aliquots, and freshly diluted in Tyrode’s solution at a final concentration of 1 μM. For combination treatments, NS5806 was applied first. After stabilization and BL pacing, hearts were perfused with NS5806 (*n* = 20). Additional drug studies followed (*n* = 5 each): VP, 2.5 μM, and SEA 1 μM. Final concentrations of 5, 15, and 45 μM NS5806 and 1 μM SEA were obtained by cumulative adding of aliquots of corresponding stock solutions 30 mM NS5806 and 2 mM SEA to Tyrode’s solution to limit the final concentration of dimethyl sulphoxide to an approximate maximum of 0.48% v/v. At BL, dimethyl sulphoxide (up to 0.5% v/v) alone produced no significant effect on mechanical and electrophysiological variables of the heart preparations.

### Data Analysis

Optical mapping data were analyzed with custom software using spatial and temporal filtering. The amplitude of the AP was normalized during processing. The peak amplitude became the maximum value and the trough became the minimum value. The AP duration, however, should not be significantly impacted by this process. AP duration was measured at 50% (APD50) and at 80% (APD80) of repolarization. Ca transient decay constant (TAU) was calculated by fitting the Ca fluorescent signal to an exponential curve. According to the data distribution, the Mann-Whitney rank sum test or Student’s *t* test, Chi-square test, or Fisher’s exact test, using SigmaStat 3.1 (Systat Software Inc., San Jose, CA), was used to compare the median and interquartile range or mean and standard deviation values of parameters and occurrence of ectopy, before and after application of NS5806, and before and after application of additional drugs. *p* < 0.05 was considered statistically significant.

## Results

We employed 5, 15, and 45 μM NS5806 to Langendorff-perfused whole rabbit hearts. Overall, we found that 5 μM NS5806 neither changed ECG morphology, nor the characteristics of APD or Ca transient. By increasing the concentration to 15 μM, minor ECG changes were induced, but not consistently. By further increasing the concentration to 45 μM, we consistently observed major ECG changes and prolonged APD.

We did not find any loss of AP dome, spontaneous sustained arrhythmias, phase-2 reentry or otherwise using any of the aforementioned doses of NS5806 in this whole heart preparation.

### NS5806 Effects on ECG

ECGs were recorded continuously at BL and during NS5806 infusion in 20 hearts. Using each rabbit heart as its own control, at 45 μM NS5806, we observed QRS widening from 60 (52, 65) to 108 (101, 146) ms, *p* < 0.001; ST-segment elevation from 0.1 (0, 0.3) to 0.3 (0.2, 0.5) mV, *p* = 0.016; and prolongation of PR interval from 88 (81, 92) to 129 (126, 177) ms, *p* < 0.001, (Figure 1C).

Spontaneous arrhythmias did not occur at BL in any of the animals. After applying NS5806, 100% of them had spontaneous premature ventricular complexes (PVCs), 55% had bigeminy, 15% had spontaneous (non-paced) T-wave alternans, 35% had ventricular tachycardia (VT) episodes, and 30% had BrS-like ECG features. All were non-sustained. Bigeminy was characterized by each sinus rhythm beat being followed by an ectopic beat with broad QRS complexes for more than 3 cycles. VT episodes were characterized by three or more consecutive ventricular ectopic beats with a rapid heart rate (> 150 bpm). No episodes of spontaneous VF were observed at BL or after NS5806 treatment. Figure 1B shows representative examples of ECG changes induced by NS5806.

### NS5806 Effects on Action Potential and Ca Transients

**Figure 2:**
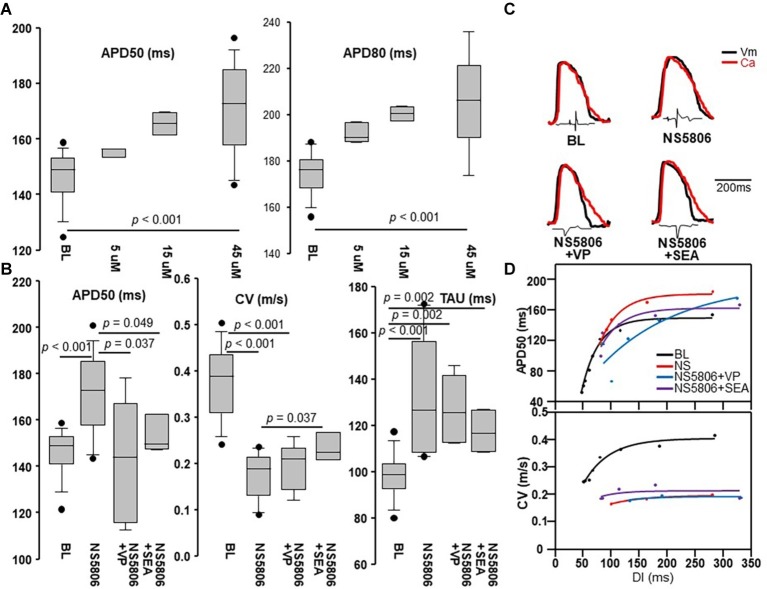
Effects of NS5806 and additional drugs on action potential (AP) and Ca transient dynamics. **(A)** APD50 (at 50% of repolarization) and APD80 at BL, 5, 15, and 45 μM of NS5806. At 45 μM, both APD50 and APD80 were prolonged. **(B)** APD50, conduction velocity (CV), and Ca exponential decay (TAU) during pacing at 3 Hz in different conditions. NS5806 significantly increased APD50, reduced CV, and increased TAU. NS5806 + SEA significantly reduced APD50 and increased CV compared to NS5806. NS5806 + VP reduced APD50 compared to NS5806. *n* = 20 animals in BL and NS5806 group, *n* = 5 animals in NS5806 + VP and NS5806 + SEA group. Box = 25th and 75th percentiles. Bars = minimum and maximum values. Dots = outliers. *p* < 0.05 was considered statistically significant. **(C)** Superimposed AP and Ca tracings during pacing at 3 Hz in different conditions. **(D)** APD50 and CV restitution in different conditions. NS5806 reached longer range of DI with an APD restitution slope > 1. NS5806 failed to maintain 1:1 capture at shorter DIs. NS5806 dropped CV by half and led to a nearly flat CV restitution curve. DI, Diastolic interval. Other abbre*via*tions as in Figure 1.

The application of 5 μM NS5806 did not change either APD50 or APD80. By increasing the concentration to 45 μM, both APD50 and APD80 were prolonged (Figure 2A). At 3 Hz pacing, NS5806 significantly increased APD50 from 146.3 ± 9.6 to 171.0 ± 16.5 ms, (*p* < 0.001) and significantly reduced conduction velocity (CV) from 0.38 ± 0.08 to 0.18 ± 0.05 m/s (*p* < 0.001). Ca transient changes were also present. NS5806 increased TAU from 98.9 (92.5, 103.6) to 126.6 (108.3, 156.6) ms, *p* < 0.001 (see Figure 2B). Figure 2C illustrates examples of the effect of NS5806 on APs and Ca transients.

### Action Potential Duration and Conduction Velocity Restitution

NS5806 dramatically changed the rate-dependence of APD and CV. Figure 2D compares APD restitution curves measured at BL and after NS5806 treatment. Driven by APD prolongation, NS5806 reached a longer range of diastolic intervals (DIs) with an APD restitution slope > 1, from 65 (56, 74) to 90 (85, 96) ms, *p* < 0.001. The maximal APD restitution slope was higher at BL. NS5806 decreased maximal pacing frequency with preserved 1:1 capture. Thus, NS5806 failed to maintain 1:1 capture at shorter DIs, increasing the DI values at which the slope of APD restitution was >1.

NS5806 dropped CV by half and led to a nearly flat CV restitution curve, in part due to lack of capture at short DIs.

**Figure 3:**
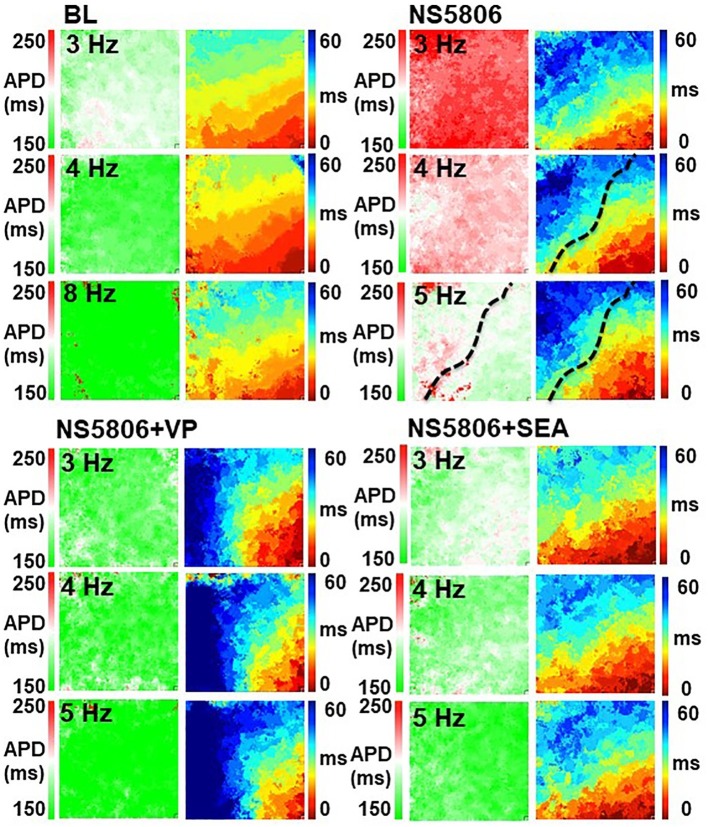
APD and isochronal maps of paced propagations under different conditions. Spatial variations in APD and CV occurred after infusion of NS5806, particularly at high pacing frequency, which were modulated by the addition of other drugs. This heterogeneous phenomena were augmented by VP and mitigated by SEA. At faster rates, CV slowing and APD prolongation became more apparent in the RV (to the right of the dotted line that represents the left anterior descending artery). Of note, APD maps are shown in same time scale to appreciate the APD prolongation with color.

Spatial variations in APD and CV occurred after infusion of NS5806, particularly at high pacing frequency, which were modulated by the addition of other drugs. These heterogeneous phenomena were augmented by VP and mitigated by SEA. Figure 3 shows APD and isochronal maps of paced propagations under different conditions. At faster rates, CV slowing and APD prolongation became more apparent in the RV (to the right of the dotted line that represents the left anterior descending artery).

### Pause-Dependent Action Potential Duration Shortening

**Figure 4:**
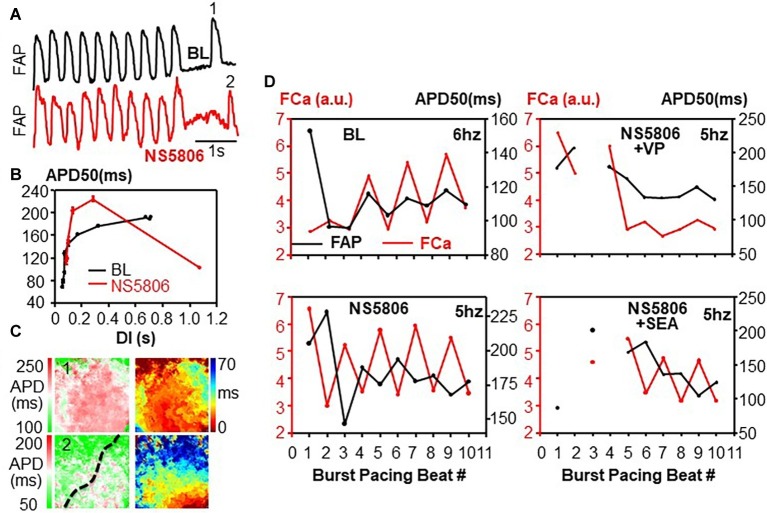
APD rate adaptation. An example of pause-dependent APD shortening during NS5806 infusion **(A)**, APD restitution curves **(B)**, and APD and isochronal map **(C)** for long DI beats (beat 1 at BL and beat 2 after NS5806). Dotted line marks location of left anterior descending artery. During NS5806 infusion, APD shortening – rather than prolongation – was noted following very long (>1 s) pauses after pacing bursts. **(D)** Phase diagrams of Ca and APD at BL and under different drugs. In BL control conditions, the first paced beat following a long DI had a long APD. Subsequent paced beats led to APD and Ca alternans, most commonly in phase (AP/Ca concordant). During NS5806 infusion, the first paced beat following a long DI had a short APD, while the Ca transient had normal rate-dependence and was large after long DIs. Thus, the short first APD had still a large Ca transient, which led to APD/Ca alternans discordance after NS5806 administration. NS5806-induced AP/Ca-discordant alternans was unaffected by SEA, but was eliminated by VP.

APD restitution curves as described above were constructed using steady-state APDs during pacing at different rates. However, during NS5806 infusion, APD shortening – rather than prolongation – was noted following very long (>1 s) pauses after pacing bursts, and after PVCs. Figure 4A shows an example. Figure 4B shows APD restitution curves and Figure 4C shows APD and isochronal map for long DI beats (beat 1 at BL and beat 2 after NS5806 in Figure 4A). This finding led us to investigate the APD rate adaptation during abrupt pacing bursts at different frequencies. In BL control conditions, the first paced beat following a long DI had a long APD. Subsequent paced beats in control conditions led to APD and Ca alternans, most commonly in phases (AP/Ca concordant). After NS5806 infusion, the first paced beat following a long DI had a short APD, while the Ca transient had normal rate-dependence and was large after long DIs. Thus, the short first APD still had a large Ca transient, which led to APD/Ca alternans discordance after NS5806 administration. Phase diagrams of Ca and APD at BL and under different drugs are shown in Figure 4D. This phenomenon was unaffected by SEA, but was eliminated by VP.

### Action Potential and Ca Alternans

**Figure 5:**
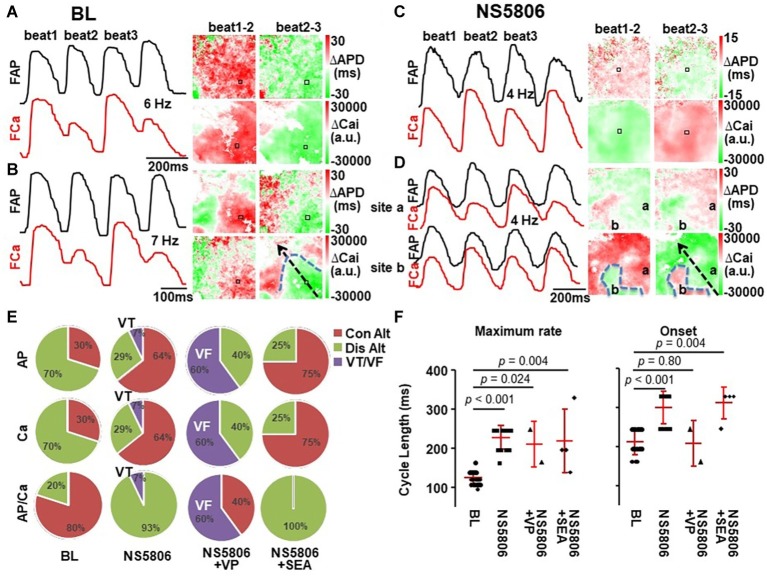
Alternans dynamics. At BL, pacing-induced alternans was predominantly electro-mechanically (AP/Ca) and spatially concordant **(A)**. Faster pacing made alternans spatially discordant with a nodal line (blue dotted line) perpendicular to propagation (black arrow) **(B)**. After NS5806 infusion, alternans was AP/Ca discordant from its onset **(C)**. Faster pacing made alternans spatially discordant with a nodal line not following propagation direction **(D)**. **(E)** The occurrence of alternans and its spatial and AP/Ca concordance at maximum capturing pacing rate. Con Alt, concordant alternans; Dis Alt, discordant alternans. **(F)** The cycle length of alternans at onset and maximum capturing pacing rate in different conditions. Lines show mean ± SD. *p* < 0.05 was considered statistically significant.

In control conditions, there was no alternans during spontaneous beating. During incremental pacing, AP and Ca alternans developed at an onset pacing cycle length (CLonset) of 200 (200, 250) ms and continued at shorter pacing cycle lengths. At the minimum capturing cycle length (CLmin) of 125 (111, 143) ms, alternans was induced at BL in all 20 hearts, of which 14 were spatially discordant and 4 were AP/Ca discordant. Thus, under control conditions, spatially discordant alternans appeared, as expected, at faster pacing rates and followed the spatial alternans phase gradients determined by the propagation direction –with the nodal line separating out-of-phase regions roughly orthogonal to propagation – and was predominantly AP/Ca concordant (see Figures 5A,B).

NS5806 facilitated the occurrence of alternans, which increased the CLonset from 200 (200, 250) to 333 (250, 333) ms, *p* < 0.001, and made alternans AP/Ca discordant from its onset (see Figure 5C). NS5806 increased CLmin from 125 (111, 143) to 250 (200, 250) ms, *p* < 0.001 (Figure 5D). At CLmin, the spatially discordant alternans arose, and was always AP/Ca discordant. Its spatial distribution occurred in patches of out-of-phase areas that did not follow the expected location determined by the propagation direction (Figure 5D). Figure 5 shows examples of AP/Ca concordant, spatially concordant (Figure 5A), spatially discordant (Figure 5B) alternans at BL and AP/Ca discordant, spatially concordant (Figure 5C), and spatially discordant (Figure 5D) alternans after NS5806 treatment. The occurrence of alternans and its spatial and AP/Ca concordance at CLmin was quantified and is shown in Figure 5E. The CL of alternans at onset and at maximum capturing pacing rate in different conditions is summarized in Figure 5F.

**Figure 6:**
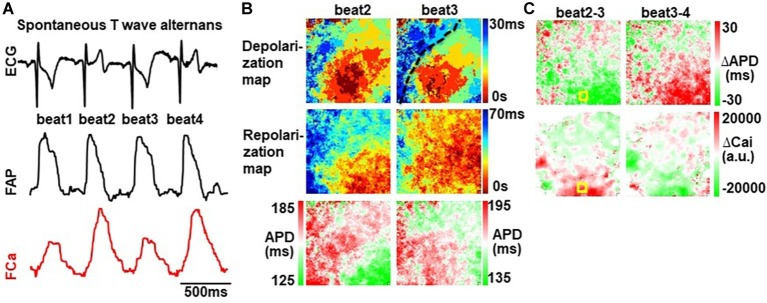
Spontaneous NS5806-induced alternans is AP/Ca and spatially discordant. **(A)** ECG of spontaneous T-wave alternans (**top**), long-short-long-short APD alternans (**mid**), and small-large-small-large Ca transient amplitude alternans (**bottom**). **(B)** Depolarization isochronal maps show a minor alternation in propagation velocity (particularly towards the RV), but the repolarization sequence was dramatically different in alternating beats. Dotted line marks location of left anterior descending artery. **(C)** Alternans maps show AP/Ca and spatial discordance.

Spontaneous (non-paced) alternans occurred only after NS5806 infusion in 4 out of the 20 hearts. AP/Ca maps showed that such alternans was both spatially and AP/Ca discordant (Figure 6C). There was a minor alternation in propagation velocity (particularly towards the RV), but the repolarization sequence was dramatically different in alternating beats (Figure 6B). Of note, spontaneous non-paced alternans did not lead to spontaneous sustained arrhythmias.

### NS5806-Induced Arrhythmias

**Figure 7:**
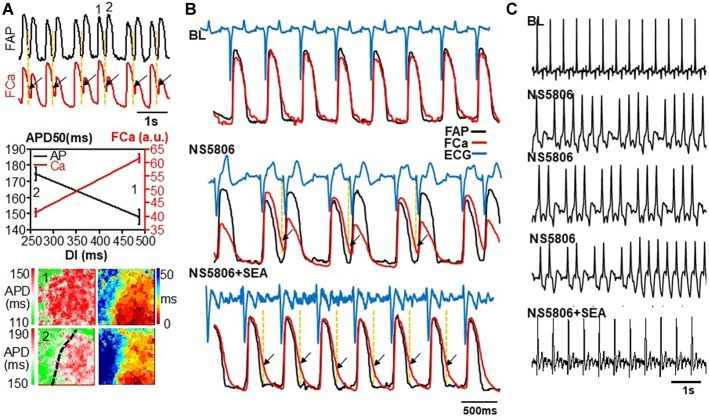
Examples of SEA suppresses NS5806-induced ectopy. **(A)** NS5806-induced spontaneous PVCs with frequent bigeminy. AP and Ca tracing during NS5806 infusion (**top**), APD and Ca versus DI (**mid**), and APD and isochronal map (**bottom**) for beat 1 and beat 2 as indicated in AP tracing (**top**). Long DIs were followed by short APDs. However, the amplitude of the Ca transient of these beats was consistently large. Ectopic beats initiated at sites in which the Ca transient was still on-going (~30.9% of maximum amplitude) while the AP had already terminated. Dotted line marks location of left anterior descending artery. Of note, APD maps are shown in different time scale to appreciate the spatial distribution with color. **(B,C)** SEA abolished NS5806-induced ventricular bigeminy and ectopic VT. Arrows indicate the high Ca load sites. Ectopic beats initiated with Ca-driven phase-4 depolarization *via* NCX. NCX blockade with SEA eliminated phase-4 depolarization.

NS5806 induced 13 non-sustained VTs, 1 sustained VT, and 1 non-sustained VF, with pacing in total of 20 hearts. However, all hearts developed spontaneous PVCs with frequent bigeminy (Figure 7A). In all cases of bigeminy, pause-dependent APD shortening appeared to play a role. At slow (spontaneous) cycle lengths, long DIs were followed by short APDs. However, the amplitude of the Ca transient of these beats was consistently large. Ectopic beats initiated at sites in which the Ca transient was still on-going (~30.9% of maximum amplitude) while the AP had already terminated (indicated with arrow in Figure 7). Although a reverse APD rate-dependence was present across the mapped tissues, sites at the PVC origin had consistently >25% of the maximum Ca transient amplitude at the onset of the PVC. Spontaneous non-sustained VT had identical mechanisms and consistently appeared focal in origin as the PVCs.

### Effects of Na-Ca Exchange in NS5806-Induced Arrhythmogenesis

Na-Ca exchange (NCX) mediates one form of AP-Ca coupling, whereby one intracellular Ca^2+^ ion is exchanged with three Na^+^ ions, leading to one net positive change entry. We hypothesized that NCX played a role in the generation of ectopic bigeminal beats. In this scenario, a large or prolonged Ca transient persisting beyond the termination of the AP would lead to electrogenic NCX and promote phase-4 depolarization *via* NCX. Five rabbit hearts underwent sequential perfusion with NS5806 followed by SEA, a NCX blocker. NS5806 + SEA reduced QRS widening from 108 (101, 146) to 73 (56, 124) ms, *p* = 0.048 and led to a ST elevation of 0.28 (0.14, 0.34) mV, which was not significantly different compared to the BL condition of 0.1 (0, 0.3) mV, *p* = 0.17. NS5806 + SEA increased prolongation of PR interval from 88 (81, 92) to 166 (132, 202) ms, *p* = 0.002. At 3 Hz pacing, NS5806 + SEA significantly reduced APD50 from 171.0 ± 16.5 to 153.3 ± 8.3 ms (*p* = 0.049), increased CV from 0.18 ± 0.05 to 0.23 ± 0.03 m/s (*p* = 0.037), and increased TAU from 98.9 (92.5, 103.6) to 116.6 (108.6, 127.0) ms, *p* = 0.002 (see Figure 2 for details). The APD prolongation induced by NS5806 was reduced, suggesting that some of the NS5806-induced APD prolongation might have been related to NCX.

NS5806 + SEA increased the CLonset of alternans from 200 (200, 250) to 333 (270, 333) ms, *p* = 0.004, and CLmin from 125 (111, 143) to 200 (200, 300) ms, *p* = 0.002. Alternans was AP/Ca discordant from its onset. At CLmin, alternans was induced in all cases, of which 25% was spatially discordant and all cases were AP/Ca discordant – unchanged from NS5806-induced alternans.

During spontaneous beating, SEA abolished NS5806-induced ventricular bigeminy and ectopic VT in all hearts (see Figure 7 examples of ECG, Ca, and AP tracing), supporting the above contention that NCX played a role in the generation of ectopic bigeminal beats.

### Effects of *I*_Ca,L_ Blockade in NS5806-Induced Arrhythmogenesis

*I*_Ca,L_ mediates AP/Ca coupling. Intracellular Ca can impact the AP *via* Ca-induced inactivation of *I*_Ca,L_, which shortens the AP. The balance between Ca-induced *I*_Ca,L_ inactivation (which shortens the AP-“negative coupling”) and Ca-mediated NCX (which prolongs the AP-“positive coupling”) is a key factor determining whether alternans is AP/Ca concordant or discordant (Sato et al., 2006). In the absence of a pharmacological means to probe Ca-induced *I*_Ca,L_ inactivation, we tested the effects of VP as an all-over *I*_Ca,L_ inhibitor. NS5806 + VP reduced QRS widening from 108 (101, 146) to 95 (51, 110) ms, *p* = 0.048 and increased ST elevation from 0.1 (0, 0.3) to 0.42 (0.21, 0.6) mV, *p* = 0.049. At 3 Hz pacing, NS5806 + VP reduced APD50 from 171.0 ± 16.5 to 142.1 ± 27.1 ms, *p* = 0.037; decreased CV from 0.38 ± 0.08 to 0.19 ± 0.05 m/s, *p* < 0.001; and increased TAU from 98.9 (92.5, 103.6) to 125.6 (112.5, 142.0) ms, *p* = 0.002.

NS5806 + VP normalized CLonset of alternans to 208 (167, 250) ms, which is no different from control condition of 200 (200, 250) ms, *p* = 0.80, and increased CLmin from 125 (111, 143) to 208 (167, 250) ms, *p* = 0.024. Alternans was induced in two-fifths of the hearts, both cases were spatially discordant but AP/Ca concordant, as opposed to the effects of NS5806 alone (see Figure 6A). Adding VP to NS5806 did not have an effect on spontaneous ventricular ectopy (VE) but it significantly increased the inducibility of VF with pacing from 7 (1/14) to 60% (3/5), *p* = 0.04 (Figure 5E).

## Discussion

The salient results of our studies are that NS5806 leads to: (1) steady-state APD prolongation with pause-dependent APD shortening; (2) CV slowing; (3) prolongation of the Ca decay constant; (4) facilitation of AP/Ca-discordant alternans, both spontaneously and pacing-induced; and (5) ventricular ectopic beats consistent with Ca-driven phase-4 depolarization *via* NCX but absence of phase-2 reentry. Additional drug testing sheds some light onto the potential mechanisms. NCX blockade with SEA eliminated phase-4 depolarization and corrected some of the NS5806-induced APD changes but did not affect alternans. VP eliminated alternans AP/Ca discordance, but increased VF inducibility at slow rates. Our data support Ca-cycling dynamics playing a role in arrhythmogenesis associated with NS5806, but do not support a reproduction of BrS phenotype by it.

### *I*_to_ Modulation by NS5806: Dose Response

A prior canine study found that 10 μM NS5806 significantly increased the magnitude of *I*_to_ and slowed inactivation in ventricular mid-myocardial cells (Calloe et al., 2009). Another study investigated the effects of different doses of NS5806 on rabbit ventricular *I*_to_. They found that10 μM NS5806 increased *I*_to_. However, 30 and 100 μM NS5806 caused a biphasic response in *I*_to_ with an initial increase followed by a decrease. These effects were not reversible with washout (Cheng et al., 2017).

Multiple modulation mechanisms of NS5806 on *I*_to_ may underlie these concentration-dependent effects seen in different studies using different animal models.

### NS5806 Effects: Conduction Velocity Slowing and Action Potential Duration Prolongation and Relation to Brugada Syndrome Arrhythmogenesis

CV slowing was a prominent effect of NS5806, and was responsible for the QRS prolongation of the electrocardiogram. It appears counterintuitive that modulating a repolarizing current would affect CV, which decreased by ~50% upon NS5806 infusion. It is conceivable that, given its timing at phase 1 of the AP, early and enhanced *I*_to_ activation may counteract the depolarizing effects of *I*_Na_, thus decreasing the slope of phase 0, its dV/dt, and therefore compromising CV. However, the CV slowing may also be due to the possibility of the off-target weak inhibitory effects of NS5806 on *I*_Na_ as reported by Calloe et al. (2009).

Overall, we observed that NS5806 led to APD prolongation during steady-state pacing at physiological rates. Previous publications have reported a bimodal response of the APD to *I*_to_ effects: either prolonging or abruptly shortening the APD (Calloe et al., 2009). *I*_to_ is normally responsible for the phase-1 notch of the AP and its “spike and dome” morphology. It has been postulated that in cells with higher *I*_to_ density, its repolarizing effects can lead to APs too low for sufficient *I*_Ca,L_ activation and to extreme AP shortening (no phase 2) (Antzelevitch, 2004). Heterogeneous APD shortening is central to the mechanistic paradigm of phase-2 reentry in BrS (Calloe et al., 2009). However, there is only one report in which epicardial and endocardial monophasic AP recordings were taken simultaneously in one patient with BrS. Transmural gradient of AP between epicardium and endocardium was observed, but shortening of AP was not reported (Kurita et al., 2002). The loss of AP dome at some epicardial sites observed in ventricular wedge preparations may not translate to the intact heart. In fact, gradients of activation recovery interval were larger in wedge than in the intact Langendorff-perfused heart, which supports artifactual AP gradients created by the wedge preparation (Boukens et al., 2017). We show that NS5806, neither alone nor in combination with VP, can reproduce BrS arrhythmogenesis in whole rabbit hearts. Thus, other mechanisms besides *I*_to_ enhancement must play a role. Mapping studies in humans with BrS (Morita et al., 2008; Nagase et al., 2008) have not observed this APD shortening, and neither did we. In computer simulations, only extreme degrees of *I*_to_ enhancement combined with extreme degrees of *I*_Na_ decrease were able to reproduce shortened APD (Zhang et al., 2015). Previous studies with the combination of AP recording and voltage-clamp experiments determined the physiological role of *I*_to_ in the rabbit crista terminalis. At physiological heart rates, the kinetics of *I*_to_ suggest that it is modulated in a frequency-dependent fashion, and therefore can contribute to the frequency-dependent effects on APD. Only under conditions where the diastolic membrane potential is sufficiently negative and the diastolic interval is long enough for the inactivation of *I*_to_ to be removed, the subsequent APD is shortened (Giles and van Ginneken, 1985). In the Ohara-Rudy AP model, the more common effect of *I*_to_ was APD prolongation for a wide range of degrees of *I*_to_ level (Zhang et al., 2015). Hoogendijk et al. have proposed that source-sink mismatch and structural heterogeneities may be important in leading to BrS ECG features and arrhythmogenesis, as clinical data seem to support (Coronel et al., 2005; Hoogendijk et al., 2010; Nademanee et al., 2015).

All studies reporting APD shortening by NS5806 consistently used unphysiologically slow rates, such as 1-s (Calloe et al., 2011) or 2-s pacing (Cheng et al., 2017). Although we found APD prolongation during steady-state pacing, with physiological APD rate-dependence and APD restitution curves (Figure 2), we did also show bradycardia-induced mild APD shortening during non-steady state conditions, most commonly, during bigeminal ventricular extrasystoles, or upon either initiation or termination of rapid pacing. During bigeminy (Figure 7), the first beat (normal) had a longer DI and a large Ca transient, and yet had a short APD. The extrasystole that followed had a much shorter DI, yet a longer APD. Interestingly, a similar behavior was shown in induced-pluripotent stem cell-derived myocytes from patients with BrS (see Figure 5 of Liang et al., 2016). We first noticed an unusually short APD in the first spontaneous beat following a very long DI (>1 s) after a rapid pacing burst. Additionally, the first paced beat of a pacing burst had shorter APD than the subsequent beats, despite its longer DI. *I*_to_ has characteristically slow recovery kinetics and it is likely that upon long DIs, its relative contribution to repolarization might have been enhanced, leading to relative APD shortening.

### *I*_to_ Modulation and Ca Cycling: Implications for Discordant Alternans

*I*_to_ has complex effects on AP/Ca coupling (Sah et al., 2003). The phase-1 portion of the AP strongly modulates the time course and magnitude of the Ca transient through its effects on trans-sarcolemmal Ca influx through *I*_Ca,L_ channels and NCXs (Sah et al., 2003). The effects of *I*_to_ modulation on the Ca transient vary among species, myocardial regions, and pathological conditions, (Sah et al., 2003) but are mediated by modulations on *I*_Ca,L_ (Sah et al., 2002). Progressive decreases in *I*_to_ and in early repolarization lead to systematic decreases in amplitude and increases in duration *I*_Ca,L_ in myocytes from rats to rabbits to guinea pigs (Linz and Meyer, 2000). Although detailed studies of *I*_Ca,L_ under conditions of *I*_to_ enhancement have not been published, it is conceivable to expect opposite effects, i.e., increased amplitude and decreased duration of *I*_Ca,L_. This, in turn, could lead to an increased amplitude of the Ca transient. Given the slow recovery kinetics of *I*_to_, longer DIs during pauses would have greater *I*_to_ currents. This phenomenon could lead to both pause-dependent mild APD shortening as well as enhancement of the Ca transient. During rapid pacing bursts, the initial beat would have a relatively longer DI and thus a short APD. The Ca transient amplitude was largest after long DIs due to both enhancement of *I*_to_ as well as the physiological Ca-cycling rate-dependence. Combined APD shortening and increased Ca transient amplitude would set the stage for the development of out-of-phase APD and Ca transient alternation.

AP/Ca discordance during alternans has been attributed to the so-called negative coupling, whereby intracellular Ca influences APD predominantly *via* Ca-induced inactivation of *I*_Ca,L_ – which would shorten APD, rather than *via* electrogenic NCX – the so-called positive coupling, which would prolong the APD (Sato et al., 2006). In this context, inhibition of NCX with SEA would further impair positive coupling, hence the lack of effect of this drug in preventing AP/Ca-discordant alternans. VP, however, eliminated AP/Ca discordant alternans. Conceivably this is the result of reduction overall of Ca entry and subsequent amplitude of the Ca transient – particularly, reduction of the pause-induced large Ca transient.

The spatial distribution of NS5806-induced spatially discordant alternans was remarkable in that it did not follow the expected pattern, where out-of-phase regions are separated by a broad nodal line, roughly perpendicular to the propagation direction. This pattern, observed at BL, is characteristic of CV restitution-mediated spatially discordant alternans, in which positive coupling predominates (Sato et al., 2006). However, after treatment with NS5806, the spatial scale of discordance followed a radically different pattern, with abrupt changes of phase in patches unrelated to the direction of propagation (Figure 5B). This finding was predicted theoretically by Sato et al. (2006) for Ca-driven alternans with negative coupling, but never confirmed experimentally before.

Alternans observed during spontaneous (non-paced) beating poses a mechanistic challenge, since it was both AP/Ca and spatially discordant. Spatial discordance cannot be explained by CV restitution, since at slow rates CV would not be compromised and the propagation of depolarization was similar for alternating beats (Figure 6B). Bradycardia-induced alternation in *I*_to_ modulation may underlie this phenomenon, whereby beats with large *I*_to_ would have larger Ca transients and shorter APDs, thus generating AP/Ca discordance. Regional differences in *I*_to_ expression may underlie spatial discordance.

### Ca-Driven Triggered Activity

The enhanced Ca transient, particularly during the short APD of long DI beats, led to triggered beats, presenting as ventricular bigeminy of focal origin, occurring after beats that had persistently elevated Ca past the APD termination. Ca concentrations of ~ 30% of peak systolic Ca after completion of the APD were present preceding bigeminal PVCs. A straightforward explanation for the triggered beats involves high Ca load leading to phase-4 depolarization *via* NCX. The triggered beat’s APD was consistently longer than that of the preceding beat, because it no longer had a long DI. The dramatic effect of SEA in eliminating ventricular bigeminy supports this contention. Focal VE has been reported in BrS (Rodriguez-Manero et al., 2016) and monomorphic extrasystoles have been shown to induce VF in BrS (Haissaguerre et al., 2003). A similar mechanism has been postulated to operate in pulmonary vein ectopy during simultaneous sympathetic and parasympathetic stimulation, which leads to increased intracellular Ca and shortened APD, respectively (Patterson et al., 2006).

### Limitations

NS5806 has been reported to have actions besides *I*_to_ opening, which are species- and tissue-dependent (Cheng et al., 2017) and may include some degree of *I*_Na_ blockade (Cheng et al., 2017). We did not use ventricular wedge preparations and, therefore, transmural propagation was not studied. This was chosen to avoid the grossly unphysiological creation of no-flux conditions and uncoupling by the transmural cut surface.

## Conclusions

NS5806 did not reproduce BrS arrhythmogenesis in intact rabbit hearts, but instead altered APD rate-dependence with steady-state APD prolongation and pause-induced APD shortening, and CV slowing. It also caused altered AP-Ca coupling, leading to AP/Ca-discordant alternans, and Ca-induced VE.

## Data Availability Statement

All datasets generated for this study are included in the article/supplementary material.

## Ethics Statement

The animal study was reviewed and approved by Houston Methodist Institutional Animal Care and Use Committee.

## Author Contributions

SW, MR-M, BK, SI-C, and LT performed the experiments and produced the data. SW analyzed optical mapping data and drafted the manuscript. LV assisted with optical mapping data analyzing. BK, SI-C, and MH analyzed ECG data. LT assisted with manuscript preparation. MV designed and supervised the overall project with the assistance of MR-M and EB, and revised and prepared the final manuscript.

### Conflict of Interest

The authors declare that the research was conducted in the absence of any commercial or financial relationships that could be construed as a potential conflict of interest.

## References

[ref1] Antzelevitch C. (2004). Cellular basis and mechanism underlying normal and abnormal myocardial repolarization and arrhythmogenesis. Ann. Med. 36(Suppl. 1), 5–14. 10.1080/17431380410032553 15176418

[ref2] Antzelevitch C. (2013). J wave syndromes: molecular and cellular mechanisms. J. Electrocardiol. 46, 510–518. 10.1016/j.jelectrocard.2013.08.006, PMID: 24011992 PMC3825797

[ref3] Bezzina C. R. Barc J. Mizusawa Y. Remme C. A. Gourraud J. B. Simonet F.. (2013). Common variants at SCN5A-SCN10A and HEY2 are associated with Brugada syndrome, a rare disease with high risk of sudden cardiac death. Nat. Genet. 45, 1044–1049. 10.1038/ng.2712, PMID: 23872634 PMC3869788

[ref4] Boukens B. J. Meijborg V. M. F. Belterman C. N. Opthof T. Janse M. J. Schuessler R. B.. (2017). Local transmural action potential gradients are absent in the isolated, intact dog heart but present in the corresponding coronary-perfused wedge. Physiol. Rep. 5:e13251. 10.14814/phy2.13251, PMID: 28554962 PMC5449556

[ref5] Calloe K. Cordeiro J. M. Di Diego J. M. Hansen R. S. Grunnet M. Olesen S. P.. (2009). A transient outward potassium current activator recapitulates the electrocardiographic manifestations of Brugada syndrome. Cardiovasc. Res. 81, 686–694. 10.1093/cvr/cvn339, PMID: 19073629 PMC2642600

[ref6] Calloe K. Nof E. Jespersen T. Di Diego J. M. Chlus N. Olesen S. P.. (2011). Comparison of the effects of a transient outward potassium channel activator on currents recorded from atrial and ventricular cardiomyocytes. J. Cardiovasc. Electrophysiol. 22, 1057–1066. 10.1111/j.1540-8167.2011.02053.x, PMID: 21457383 PMC3136585

[ref7] Cerrone M. Lin X. Zhang M. Agullo-Pascual E. Pfenniger A. Chkourko Gusky H.. (2014). Missense mutations in plakophilin-2 cause sodium current deficit and associate with a Brugada syndrome phenotype. Circulation 129, 1092–1103. 10.1161/CIRCULATIONAHA.113.003077, PMID: 24352520 PMC3954430

[ref8] Chen Q. Kirsch G. E. Zhang D. Brugada R. Brugada J. Brugada P.. (1998). Genetic basis and molecular mechanism for idiopathic ventricular fibrillation. Nature 392, 293–296. 10.1038/32675, PMID: 9521325

[ref9] Cheng H. Cannell M. B. Hancox J. C. (2017). Differential responses of rabbit ventricular and atrial transient outward current (Ito) to the Ito modulator NS5806. Physiol. Rep. 5:e13172. 10.14814/phy2.13172, PMID: 28270595 PMC5350179

[ref10] Coronel R. Casini S. Koopmann T. T. Wilms-Schopman F. J. Verkerk A. O. de Groot J. R.. (2005). Right ventricular fibrosis and conduction delay in a patient with clinical signs of Brugada syndrome: a combined electrophysiological, genetic, histopathologic, and computational study. Circulation 112, 2769–2777. 10.1161/CIRCULATIONAHA.105.532614, PMID: 16267250

[ref11] de Diego C. Pai R. K. Dave A. S. Lynch A. Thu M. Chen F.. (2008). Spatially discordant alternans in cardiomyocyte monolayers. Am. J. Physiol. Heart Circ. Physiol. 294, H1417–H1425. 10.1152/ajpheart.01233.2007, PMID: 18223190

[ref12] Fedorov V. V. Lozinsky I. T. Sosunov E. A. Anyukhovsky E. P. Rosen M. R. Balke C. W.. (2007). Application of blebbistatin as an excitation-contraction uncoupler for electrophysiologic study of rat and rabbit hearts. Heart Rhythm. 4, 619–626. 10.1016/j.hrthm.2006.12.047, PMID: 17467631

[ref13] Giles W. R. van Ginneken A. C. (1985). A transient outward current in isolated cells from the crista terminalis of rabbit heart. J. Physiol. 368, 243–264. 10.1113/jphysiol.1985.sp015856, PMID: 2416913 PMC1192595

[ref14] Giudicessi J. R. Ye D. Tester D. J. Crotti L. Mugione A. Nesterenko V. V.. (2011). Transient outward current (I(to)) gain-of-function mutations in the KCND3-encoded Kv4.3 potassium channel and Brugada syndrome. Heart Rhythm. 8, 1024–1032. 10.1016/j.hrthm.2011.02.021, PMID: 21349352 PMC3150551

[ref15] Haissaguerre M. Extramiana F. Hocini M. Cauchemez B. Jais P. Cabrera J. A.. (2003). Mapping and ablation of ventricular fibrillation associated with long-QT and Brugada syndromes. Circulation 108, 925–928. 10.1161/01.CIR.0000088781.99943.95, PMID: 12925452

[ref16] Holley C. L. Cooper J. A. (2009). Macrovolt T-wave alternans and polymorphic ventricular tachycardia. Circulation 120, 445–446. 10.1161/CIRCULATIONAHA.109.861633, PMID: 19652119

[ref17] Hoogendijk M. G. Potse M. Linnenbank A. C. Verkerk A. O. den Ruijter H. M. van Amersfoorth S. C.. (2010). Mechanism of right precordial ST-segment elevation in structural heart disease: excitation failure by current-to-load mismatch. Heart Rhythm. 7, 238–248. 10.1016/j.hrthm.2009.10.007, PMID: 20022821

[ref18] Kurita T. Shimizu W. Inagaki M. Suyama K. Taguchi A. Satomi K. (2002). The electrophysiologic mechanism of ST-segment elevation in Brugada syndrome. J. Am. Coll. Cardiol. 40, 330–334. 10.1016/s0735-1097(02)01964-2 12106940

[ref19] Liang P. Sallam K. Wu H. Li Y. Itzhaki I. Garg P. (2016). Patient-specific and genome-edited induced pluripotent stem cell-derived cardiomyocytes elucidate single-cell phenotype of Brugada syndrome. J. Am. Coll. Cardiol. 68, 2086–2096. 10.1016/j.jacc.2016.07.779 27810048 PMC5373649

[ref20] Linz K. W. Meyer R. (2000). Profile and kinetics of L-type calcium current during the cardiac ventricular action potential compared in Guinea-pigs, rats and rabbits. Pflugers Arch. 439, 588–599. 10.1007/s004249900212 10764219

[ref21] Liu M. B. de Lange E. Garfinkel A. Weiss J. N. Qu Z. (2015). Delayed afterdepolarizations generate both triggers and a vulnerable substrate promoting reentry in cardiac tissue. Heart Rhythm. 12, 2115–2124. 10.1016/j.hrthm.2015.06.019, PMID: 26072025 PMC4583816

[ref22] Lou Q. Li W. Efimov I. R. (2012). The role of dynamic instability and wavelength in arrhythmia maintenance as revealed by panoramic imaging with blebbistatin vs. 2,3-butanedione monoxime. Am. J. Physiol. Heart Circ. Physiol. 302, H262–H269. 10.1152/ajpheart.00711.2011, PMID: 22037192 PMC3334231

[ref23] Minoura Y. Panama B. K. Nesterenko V. V. Betzenhauser M. Barajas-Martinez H. Hu D.. (2013). Effect of Wenxin Keli and quinidine to suppress arrhythmogenesis in an experimental model of Brugada syndrome. Heart Rhythm. 10, 1054–1062. 10.1016/j.hrthm.2013.03.011, PMID: 23499631 PMC3702731

[ref24] Morita H. Zipes D. P. Fukushima-Kusano K. Nagase S. Nakamura K. Morita S. T.. (2008). Repolarization heterogeneity in the right ventricular outflow tract: correlation with ventricular arrhythmias in Brugada patients and in an in vitro canine Brugada model. Heart Rhythm. 5, 725–733. 10.1016/j.hrthm.2008.02.028, PMID: 18452878

[ref25] Morita H. Zipes D. P. Lopshire J. Morita S. T. Wu J. (2006). T wave alternans in an in vitro canine tissue model of Brugada syndrome. Am. J. Physiol. Heart Circ. Physiol. 291, H421–H428. 10.1152/ajpheart.01259.2005, PMID: 16648179

[ref26] Nademanee K. Raju H. de Noronha S. V. Papadakis M. Robinson L. Rothery S. (2015). Fibrosis, Connexin-43, and conduction abnormalities in the Brugada syndrome. J. Am. Coll. Cardiol. 66, 1976–1986. 10.1016/j.jacc.2015.08.862 26516000 PMC4631798

[ref27] Nagase S. Kusano K. F. Morita H. Nishii N. Banba K. Watanabe A. (2008). Longer repolarization in the epicardium at the right ventricular outflow tract causes type 1 electrocardiogram in patients with Brugada syndrome. J. Am. Coll. Cardiol. 51, 1154–1161. 10.1016/j.jacc.2007.10.059 18355652

[ref28] Patocskai B. Barajas-Martinez H. Hu D. Gurabi Z. Koncz I. Antzelevitch C. (2016). Cellular and ionic mechanisms underlying the effects of cilostazol, milrinone, and isoproterenol to suppress arrhythmogenesis in an experimental model of early repolarization syndrome. Heart Rhythm. 13, 1326–1334. 10.1016/j.hrthm.2016.01.024, PMID: 26820510 PMC4879023

[ref29] Patocskai B. Yoon N. Antzelevitch C. (2017). Mechanisms underlying Epicardial radiofrequency ablation to suppress Arrhythmogenesis in experimental models of Brugada syndrome. JACC Clin. Electrophysiol. 3, 353–363. 10.1016/j.jacep.2016.10.011, PMID: 28948234 PMC5609479

[ref30] Patterson E. Lazzara R. Szabo B. Liu H. Tang D. Li Y. H. (2006). Sodium-calcium exchange initiated by the Ca2+ transient: an arrhythmia trigger within pulmonary veins. J. Am. Coll. Cardiol. 47, 1196–1206. 10.1016/j.jacc.2005.12.023 16545652

[ref31] Rodriguez-Manero M. Sacher F. de Asmundis C. Maury P. Lambiase P. D. Sarkozy A.. (2016). Monomorphic ventricular tachycardia in patients with Brugada syndrome: a multicenter retrospective study. Heart Rhythm. 13, 669–682. 10.1016/j.hrthm.2015.10.038, PMID: 26538325

[ref32] Sah R. Ramirez R. J. Backx P. H. (2002). Modulation of Ca(2+) release in cardiac myocytes by changes in repolarization rate: role of phase-1 action potential repolarization in excitation-contraction coupling. Circ. Res. 90, 165–173. 10.1161/hh0202.103315, PMID: 11834709

[ref33] Sah R. Ramirez R. J. Oudit G. Y. Gidrewicz D. Trivieri M. G. Zobel C.. (2003). Regulation of cardiac excitation-contraction coupling by action potential repolarization: role of the transient outward potassium current (I(to)). J. Physiol. 546, 5–18. 10.1113/jphysiol.2002.026468, PMID: 12509475 PMC2342473

[ref34] Sato D. Shiferaw Y. Garfinkel A. Weiss J. N. Qu Z. Karma A. (2006). Spatially discordant alternans in cardiac tissue: role of calcium cycling. Circ. Res. 99, 520–527. 10.1161/01.RES.0000240542.03986.e7, PMID: 16902177

[ref35] Szel T. Antzelevitch C. (2014). Abnormal repolarization as the basis for late potentials and fractionated electrograms recorded from epicardium in experimental models of Brugada syndrome. J. Am. Coll. Cardiol. 63, 2037–2045. 10.1016/j.jacc.2014.01.067 24657694 PMC4024366

[ref36] Szel T. Koncz I. Antzelevitch C. (2013). Cellular mechanisms underlying the effects of milrinone and cilostazol to suppress arrhythmogenesis associated with Brugada syndrome. Heart Rhythm. 10, 1720–1727. 10.1016/j.hrthm.2013.07.047, PMID: 23911896 PMC3825770

[ref37] Uchimura-Makita Y. Nakano Y. Tokuyama T. Fujiwara M. Watanabe Y. Sairaku A.. (2014). Time-domain T-wave alternans is strongly associated with a history of ventricular fibrillation in patients with Brugada syndrome. J. Cardiovasc. Electrophysiol. 25, 1021–1027. 10.1111/jce.12441, PMID: 24761970

[ref38] Yan G. X. Antzelevitch C. (1999). Cellular basis for the Brugada syndrome and other mechanisms of arrhythmogenesis associated with ST-segment elevation. Circulation 100, 1660–1666. 10.1161/01.CIR.100.15.1660, PMID: 10517739

[ref39] Yoon N. Patocskai B. Antzelevitch C. (2018). Epicardial substrate as a target for radiofrequency ablation in an experimental model of early repolarization syndrome. Circ. Arrhythm. Electrophysiol. 11:e006511. 10.1161/CIRCEP.118.006511, PMID: 30354293

[ref40] Zhang J. Sacher F. Hoffmayer K. O’Hara T. Strom M. Cuculich P.. (2015). Cardiac electrophysiological substrate underlying the ECG phenotype and electrogram abnormalities in Brugada syndrome patients. Circulation 131, 1950–1959. 10.1161/CIRCULATIONAHA.114.013698, PMID: 25810336 PMC4452400

